# Soft Conductive Textile Sensors: Characterization Methodology and Behavioral Analysis

**DOI:** 10.3390/s25144448

**Published:** 2025-07-17

**Authors:** Giulia Gamberini, Selene Tognarelli, Arianna Menciassi

**Affiliations:** 1 Health Science Interdisciplinary Center, Scuola Superiore Sant’Anna, 56124 Pisa, Italy; giulia.gamberini@santannapisa.it; 2 The BioRobotics Institute, Scuola Superiore Sant’Anna, 56025 Pontedera, Italy; 3 The Department of Excellence in Robotics & AI, Scuola Superiore Sant’Anna, 56127 Pisa, Italy

**Keywords:** resistive stretching sensor, soft sensors, conductive fabrics, sensors’ mechanical characterization, electrical properties

## Abstract

Resistive stretching sensors are currently used in healthcare robotics due to their ability to vary electrical resistance when subjected to mechanical strain. However, commercial sensors often lack the softness required for integration into soft structures. This study presents a detailed methodology to characterize fabric-based resistive stretching sensors, focusing on both static and dynamic performance, for application in a smart vascular simulator for surgical training. Five sensors, called #1–#5, were developed using conductive fabrics integrated into soft silicone. Stability and fatigue tests were performed to evaluate their behavior. The surface structure and fiber distribution were analyzed using digital microscopy and scanning electron microscopy, while element analysis was performed via Energy-Dispersive X-ray Spectroscopy. Sensors #1 and #3 are the most stable with a low relative standard deviation and good sensitivity at low strains. Sensor #3 showed the lowest hysteresis, while sensor #1 had the widest operating range (0–30% strain). Although all sensors showed non-monotonic behavior across 0–100% strain, deeper investigation suggested that the sensor response depends on the configuration of conductive paths within and between fabric layers. Soft fabric-based resistive sensors represent a promising technical solution for physical simulators for surgical training.

## 1. Introduction

Conductive textiles can be used to realize resistive stretching sensors, which are characterized by a change in electrical resistance as a function of the applied mechanical strain. Commercial and conventional sensor technologies, e.g., Force Sensing Resistor by Interlink Electronics Inc., (Camarillo, CA, USA) [1] and FlexiForce sensors by Tekscan Inc. (Providence Highway Norwood, MA, USA) [2], are usually not soft enough for many applications. Consequently, shaping them to fit soft structures is not always feasible due to stiffness constraints. The lack of customizable, commercial, flexible sensors characterized by lightweight design, ease of integration, and ease of application has increased the research interest in stretchable and flexible sensors since the beginning of the 21st century [3]. Despite recognized achievements, the adoption of flexible sensors is still very limited [4].

Flexible sensors offer a broad spectrum of applications within healthcare robotics. These range from body monitoring [5] and drug release [6] to the detection of joint motion [7], respiration monitoring [8], pulse monitoring [9], the creation of soft prosthetic limbs [10], and artificial skin [11]. Their usage extends to soft robotic grippers [12], smart wearable strain sensors [13], and even into gaming [14] and virtual reality applications [15].

In the literature, flexible sensors have been obtained through various technological solutions, such as graphene [8], stretchable films with microgels [6], capacitive fibers made of ionically conductive fluid and silicone elastomer [7], graphite thin films [8], waveguides [10], nanocomposite silver nanowire [13], conductive fabrics [14], and smart textile fabrics [16]. Among these, conductive fabrics are recognized in the literature as one of the most promising technological solutions for the development of stretchable sensors [17,18]. Conductive fabrics are typically lightweight, easily integrable, and low-cost; they are based on piezoresistive fibrous materials with an electrical resistance that varies under mechanical stress [19]. This characteristic enables the measurement of their response to strain by observing the variation in resistance [17].

Conductive fabrics are widely cited in the literature and used in biomedical applications. Huang et al. presented a wearable yarn-based piezoresistive sensor to monitor and track respiratory signals [20]. Carnevale et al. developed, characterized, and tested a wearable stretchable sensor for monitoring shoulder range of motion [21]. Taji et al. presented an ECG monitoring system using conductive fabric dry electrodes [22]. Watson et al. presented a stretchable conductive fabric sensor used to measure knee angle [23].

Considering the features of conductive fabrics, as presented above, soft conductive stretching sensors could be integrated into silicone structures to realize smart, high-fidelity physical simulators for surgical training [24]. Soft sensing technologies could be used to provide an objective assessment of the trainees’ performance.

The literature presents various types of characterizations for soft conductive textile sensors, but a standardized methodology has not been widely adopted among researchers. Some studies present static mechanical characterization without considering the dynamic performance of the sensors [25]. On the other hand, other studies focus primarily on the dynamic response and fatigue performance of the sensors without any static analysis [26,27].

In this work, we propose a comprehensive methodology to characterize fabric-based resistive stretching sensors from both static and dynamic perspectives across five sensor types. The specific aim is to identify the best sensor for integration into a sensorized high-fidelity lung physical simulator for robotic lobectomy. This simulator will be used to train novices in the isolation and resection of vasculature structures in robot-assisted surgery. Moreover, based on the characterization conducted and on the obtained calibration curve, further analysis was carried out on the most promising sensor technology for our application in order to understand the underlying behavior. In addition, the few existing lung simulators [28,29,30] are not sensorized; thus, this work prompts the integration and realization of a sensorized high-fidelity lung simulator for robotic surgical training.

In the following sections, we present the Materials and Methods, along with the characteristics needed for our application and the stability and durability tests. We also explain the measured properties and include a surface analysis. In the Results Section, we provide an analysis of the performance and characteristics of the sensors. Finally, in the Section 4, we analyze the characteristics of the sensor fibers (*i*) to understand the underlying phenomena, (*ii*) to evaluate changes in the concentration of anatomical elements on the sensor surface, and (*iii*) to formulate a mathematical model that explains the sensor’s output behavior and can be applied to other sensor types.

## 2. Materials and Methods

### 2.1. Sensor Specifications for Vasculature Physical Simulator

High-fidelity physical simulators replicate patient anatomy and physiology using soft materials, such as silicone, which mimic the mechanical properties of human tissue [31]. These simulators can also be equipped with sensors to evaluate a trainee’s performance and assess their ability to carry out specific procedures. For effective integration into such simulators, sensors have to meet specific technical requirements:

- Stability: Sensor readings should be stable when stretched at a fixed strain.
- Appropriate working range: The sensor operating range should match the simulator working conditions, i.e., stretching range.
- Sensor–soft structure compatibility: When integrated into soft materials, the sensor features (dimensions, thickness, and softness) have to be compatible with its mechanical properties.
- Durability: Sensor performance should not degrade when stretched multiple times and over time.

Our specific application involves a high-fidelity physical simulator of vasculature structures for training the isolation and resection of blood vessels, a common procedure in the treatment of cancerous tissues. During these operations, blood vessels can undergo significant deformation; excessive strain may cause vessel rupture and lead to intraoperative bleeding. Therefore, it is crucial for surgeons to learn how much strain can be safely applied. To replicate this training scenario, the vessel simulator should include sensors capable of detecting strain between 0% and 30%, which is the threshold at which vessel rupture typically occurs [32], and that show high stability when stretched for 30 s. Moreover, as previously mentioned, to replicate the properties of human tissues, silicone could be employed. Therefore, the sensor should be integrated into silicone structures but not completely embedded, to avoid malfunction due to silicone getting caught in the layers of the conductive fabrics. Finally, it should be capable of undergoing repeated stretching to allow multiple training sessions.

### 2.2. Fabrics Used for Sensor Design and Development

**Figure A1:**
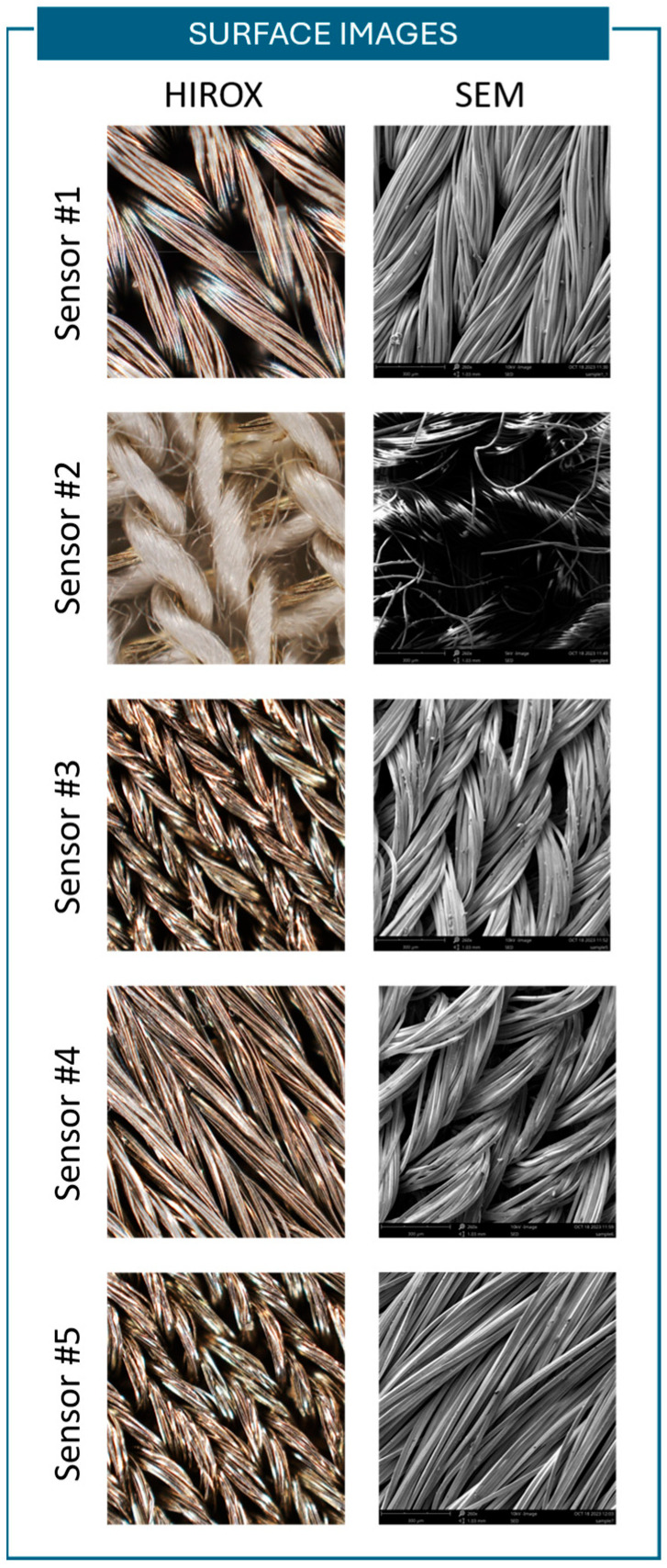
Surface images taken under a Hirox microscope (140×) and an SEM (260×) for all five sensors. As the fabrics are conductive, no prior treatment was needed.

Different customized resistive stretching sensors were created by using five commercially available conductive fabrics. The selection of these fabrics was based on a comparison of the literature results, market analysis, and sample availability. These five fabrics are reported in Table 1, along with additional details. To obtain qualitative information about the sensor surface, microscope images were taken using a Hirox HRX-01 microscope (Hirox, Tokyo, Japan), while the Scanning Electron Microscope (SEM) Phenom XL (Thermo Fisher Scientific, Waltham, MA, USA) was used to determine the dimensions of the sensor fibers. Microscope and SEM images for each sensor are shown in Figure A1 in Appendix A.

The fabric numbers, reported in the first column of Table 1, will be used in the following paragraphs for the unique sensors’ identification.

**Figure 1:**
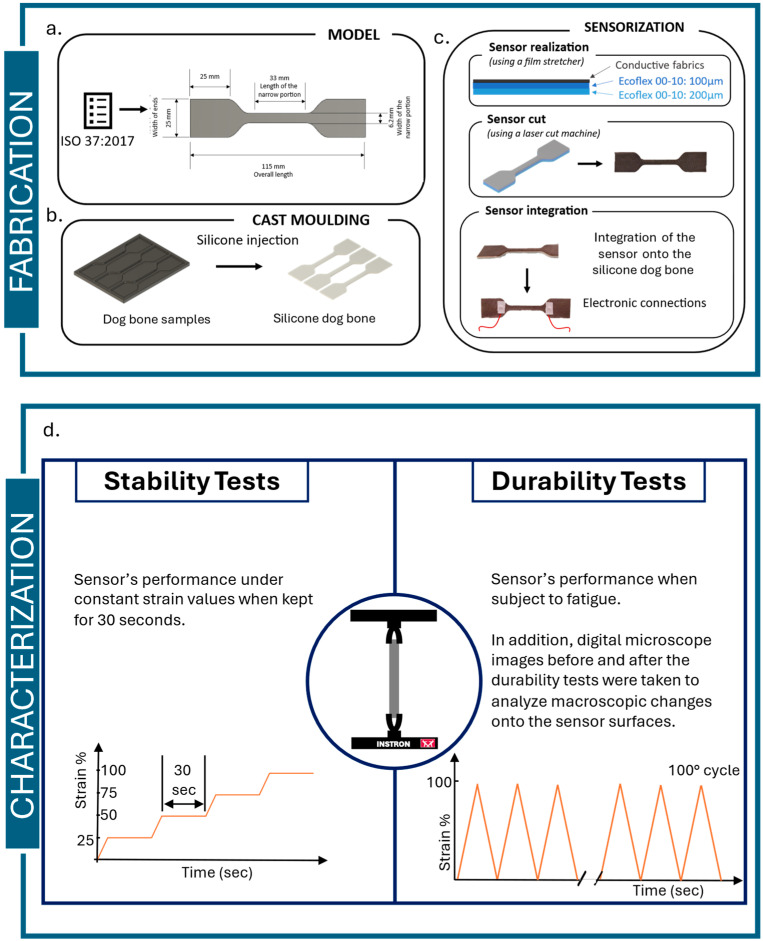
Sensors’ fabrication steps: Modeling realized following the ISO standard 37:2024 [33] (**a**), soft structure realization by cast molding (**b**), and sensorization of the soft structures (the electronic connections are depicted with red wires, while the white part is the thermoadhesive material used to fix the electronic connectors). (**c**) Characterization methodology used to analyze the sensor behaviors: stability test to evaluate the sensor stability and durability test to evaluate the sensor fatigue (**d**).

As reported in Table 1, the conductive fabrics are made of different materials, and the fiber dimensions vary by fabric typology. The conductive fabrics were used to develop five different sensors. For evaluating the sensor behavior in soft structures (e.g., soft gripper, soft simulators, soft manipulators), the sensors were obtained by combining a soft substrate and a conductive fabric. Both the soft structure and the conductive fabric shape were designed following the ISO Standard 37:2017 [33]; namely, the sensors were designed with a dog-bone or dumbbell shape, with an overall length of 115 mm and other dimensions as shown in Figure 1a.

### 2.3. Sensors’ Fabrication

The main technique used for sensor fabrication was cast molding. The mold was designed using Fusion 360 software (Autodesk, San Francisco, CA, USA) and 3D-printed with an Original Prusa i3 MK3s+ 3D printer (Prusa Research, Prague, Czech Republic) by using polyethylene terephthalate modified with glycol (PETG). To create the soft structure, silicone Ecoflex 00-30 (Smooth-On, Macungie, PA, USA) was used. Part A and part B of the silicone were mixed in a 1A:1B ratio by weight, as shown in Figure 1b. Due to its flexibility, softness, and structural integrity, Ecoflex rubber is an ideal candidate for developing the soft structure for fabricating flexible strain sensors [34]. The soft dog-bone structure was prepared, poured, and cured at room temperature for 4 h.

In the meantime, while the silicone was polymerizing, the sensing part was fabricated. Ecoflex 00-10 was used, and a first layer of 200 µm was cast onto a PETG sheet using an automatic film stretcher (TCQ Sheen B. V., Capelle Aan Den Ijssel, Zuid-Holland, The Netherlands). This first layer was polymerized in the oven at 50 °C for 15 min. Again using the film stretcher, a second layer of 100 μm thickness was cast onto the first layer. The conductive fabric was then positioned on the second layer and immediately placed into the oven at 50 °C for 30 min.

Once the silicone dog bone was polymerized, the silicone–conductive fabric structure was adhered to the surface of the dog bone using an additional 100 μm thick layer of Ecoflex 00-10. The resulting soft resistive stretching sensor was cured in the oven at 50 °C for 30 min. Signal acquisition was performed using conductive wires attached to the sensor surface with commercially available thermoadhesive material, as shown in Figure 1c.

### 2.4. Electronics and Software

To obtain reliable sensor signals, a data acquisition (DAQ) board, NI USB 6009 (National Instrument, Austin, TX, USA), and a Wheatstone bridge were used to measure the electrical resistance of the soft stretching sensors. The Wheatstone bridge was optimized by considering the resistance value of each sensor type in its resting state. Two resistances, R1 and R2, were chosen to have constant values across the different sensor types, while the third one, R3, was selected each time to balance the Wheatstone bridge in the sensor’s rest configuration. The value of R1 is 100 Ω, while R2 is 150 Ω. To control the electronics and to visualize and save the data from the different sensors, LabVIEW software (National Instrument, Austin, TX, USA) was used to implement a user-friendly Graphical User Interface (GUI).

**Table 1:** Fabrics used for sensor realization: details. (*): the fiber dimension (width) was extrapolated from SEM images. For sensor #2, it was not possible to extract a unique width dimension. As depicted in Figure A1 in Appendix A, the sensor has a particular structure which does not allow for the extraction of a width dimension for a single sensor fiber.

Fabric #	Name	Company	Composition	Fiber Dimension—Width (*)
1	Stretch Conductive Fabrics [35]	Less EMF (Latham, NY, USA)	Silver-plated, 76% Nylon, 24% Elastic fabric	12.7 µm
2	Silverell Fabrics [36]	Less EMF (USA)	16% Silver–Nylon, 84% Rayon	---
3	Technik-tex P130+B [37]	Shieldex (Bremen, Germany)	78% Polyamide, 22% Elastomer	17.3 µm
4	Technik-tex P180+B [38]	Shieldex (Germany)	94% Polyamide, 6% Dorlastan	18.2 µm
5	Med-tex P130 [39]	Shieldex (Germany)	78% Polyamide, 22% Elastomer	20.3 µm

### 2.5. Characterization Methodology: Tests Performed and Properties Measured

The sensor characterization was conducted in accordance with the literature, using an Instron machine (Instron, Norwood, MA, USA) to perform monoaxial tensile tests. The sensor data were acquired and saved by using the electronics previously described. The sample was placed between the Instron grippers, and its initial length (L0) was measured. The data acquisition sampling frequency was 1 kHz.

**Figure 2:**
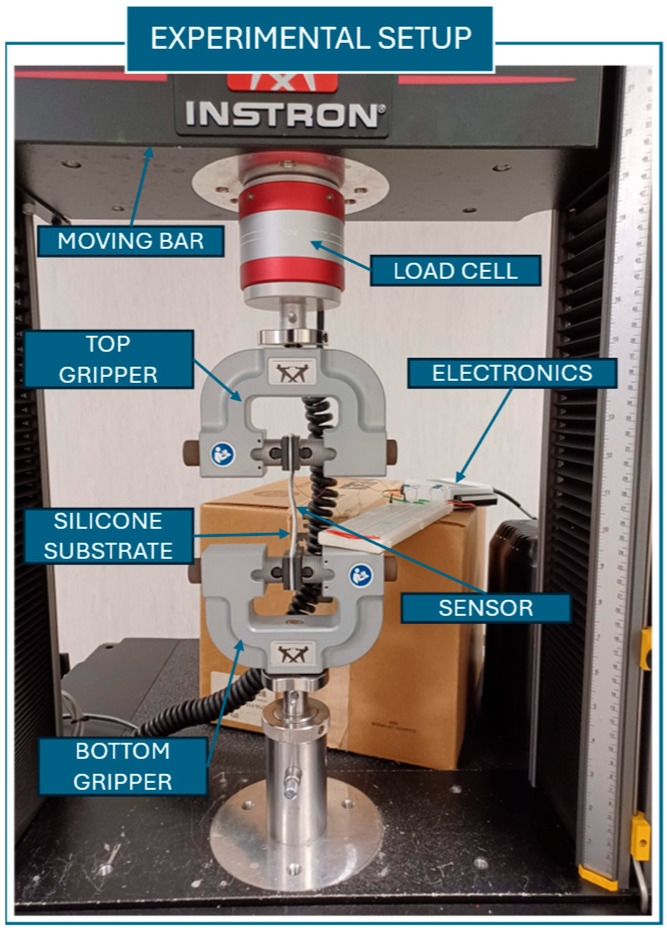
Experimental setup used to carry out the characterization test of the sensors.

In Figure 2, the experimental setup used for the different characterization tests is depicted.

Stability test

The stability test assesses the sensor’s resistance at various elongation levels, with the sample held at a specified strain for a set duration. The sensor was tested at strain levels of 25%, 50%, 75%, and 100%. To assess the sensor’s stability, a ramp–hold method was implemented using the Instron control software (Bluehill Universal for Universal Testing Systems, Instron, Norwood, MA, USA). The Instron machine was displacement-controlled, cycling between the minimum and maximum strain values, with each strain level held for 30 s.

Through the stability test, the following measured properties were calculated:

- The mean and standard deviation of the resistance value at each elongation.
- The gauge factor (GF), i.e., the relative change in resistance divided by the relative change in length (i.e., the definition of strain). The GF is calculated using (1)

(1) $$Gauge\;Factor=\frac{\frac{Rn-R0}{R0}}{\frac{Ln-L0}{L0}}\;,$$

where *Rn* indicates the resistance at N mm of elongation, *R*0 is the resistance at 0 mm of elongation (rest position), *Ln* refers to the elongation at N mm, and *L*0 is the length at rest [35].

**Figure 3:**
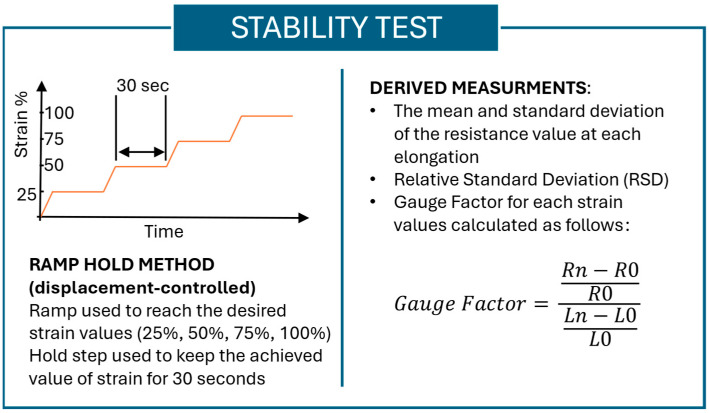
Summary of the stability test methodology and the measurements derived from the stability tests.

In Figure 3, the parameters used and properties measured in the stability test are summarized.

Durability test

The durability test evaluates sensor performance under fatigue. One hundred cyclic monoaxial tensile tests were performed, where the samples were stretched between 0% and 100% of strain at a rate of 500 mm/min. The characteristic or calibration curve of each sensor is defined by the values of Δ*R*/*R*0 at different elongation values, where Δ*R* is the difference between the current resistance value and the resistance value in the resting state. The characteristic curve is obtained by combining the sensor readings and the strain data from the Instron machine, using appropriate resampling techniques. Furthermore, the durability test allows for the evaluation of several additional sensor properties, such as the (*i*) repeatability, (*ii*) hysteresis, and (*iii*) operating range. The mentioned properties were calculated as follows:

- The characterization curve was plotted at different numbers of cycles, such as 1st cycle, 9th cycle, 19th cycle, 29th cycle, and 99th cycle. The choice of considering different cycles is due to the analysis of the behavioral change when the sensor is subjected to fatigue. For each cycle, both the upward—from 0% to 100% of strain—and downward—from 100% to 0% of strain—curves were plotted. These curves were used to evaluate the repeatability, hysteresis, and operating range of the sensors. From the characterization curve, the calibration equation of each sensor can be derived.
- Repeatability is evaluated considering the drift of the sensor. The drift is a feature of the sensor determined by repeating cycles. It describes an unwanted shift in the sensor output when the input does not change. Instead of occurring suddenly, this variation is usually noticed over long periods of time and might be attributed to age, environmental variables, or the intrinsic properties of the sensor materials [40]. In accordance with the literature, in this work, the difference between the first point of both the 2nd and the 99th cycles was taken as an indicator of drift and thus of repeatability, as in [26,27].

In particular, by considering the characterization curves of the sensors, the drift was calculated as reported below in Equation (2):

(2) $$drift={\frac{∆R}{R0}\left(99th\;cycle\right)}_{1}-{\frac{∆R}{R0}(2th\;cycle)}_{1}$$

- The hysteresis of a sensor is defined as the difference between the resistance at a given strain in the loading cycle and the resistance at the same strain in the unloading cycle for each strain value. The hysteresis error of a sensor is defined as the difference in the output at any measurement value within the sensor’s specified range, observed when the measurement point is approached first by increasing the strain and then by decreasing the strain. The maximum hysteresis error was calculated both for the 1st and the 99th cycles to evaluate the influence of fatigue on the hysteresis behavior of the sensor.

In particular, it was calculated as the maximum error between the difference in y values assumed by the upward and downward curves, as reported in Equation (3):

(3) $$hysteresis\;error=\max\left(|\frac{∆R}{R0}\left(upward\;cycle\right)-\frac{∆R}{R0}(downward\;cycle)|\right)$$

- The operating range is evaluated considering the characteristic curve of each sensor. The calibration curve obtained for each sensor was used to identify the working range of the sensors. The operating range was stated considering the region in which the sensor is monotonic.

In addition, to analyze the sensors’ surface before and after the durability test and thus to understand whether the durability test affects the surface characteristics of the sensors, microscope images were taken using a Hirox microscope.

**Figure 4:**
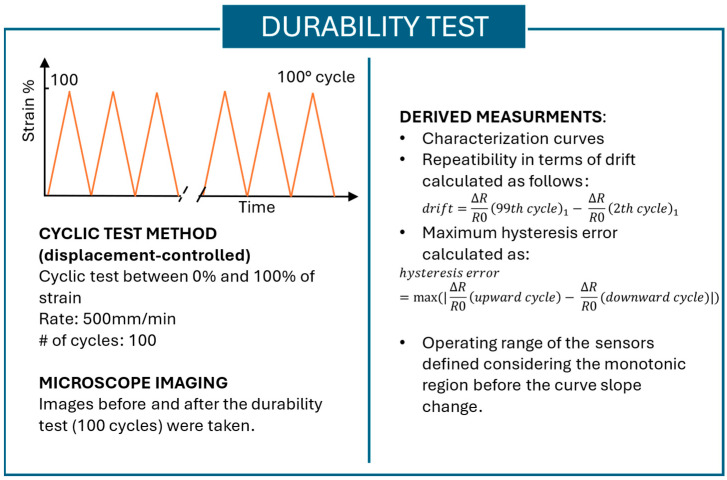
Summary of the durability test methodology and the measurements derived from the durability tests.

In Figure 4, the parameters used and properties measured in the durability test are summarized.

## 3. Results

A full characterization was carried out on the five sensor typologies. For each sensor type, three samples were fabricated and tested.

### 3.1. Stability Test

**Figure 5:**
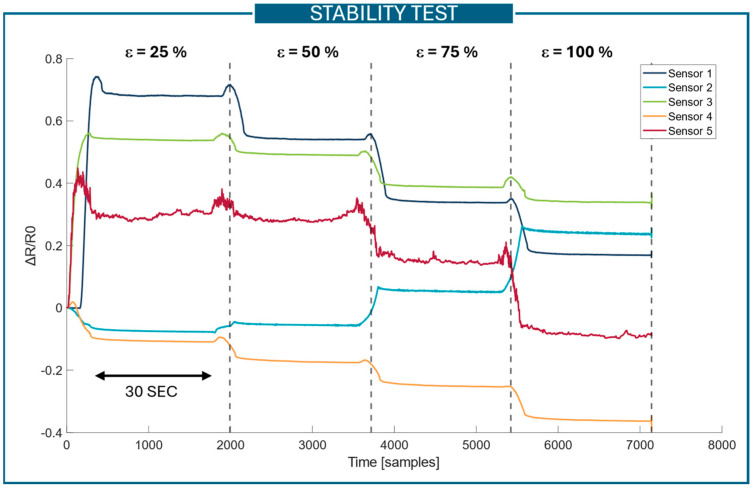
Results of the stability test: three repetitions for three samples of each sensor type were tested. The dashed lines are used to separate the different strain values at which the sensors were kept for 30 s. The sensor curves refer to the variation in resistance with respect to the rest value.

The results of the stability test are reported in Figure 5. The graph shows the behavior of the five different sensors when stretched at 25%, 50%, 75%, and 100% of strain and kept for 30 s at these strain values. The complete table containing the means and standard deviations of the resistance value for each elongation is reported in Appendix A—Table A1. To give an indication of the sensor performance in terms of stability, the relative standard deviation (RSD) was calculated. Sensor #1 and sensor #3 are the ones showing better performance in terms of stability; they have an RSD of between 0.4% and 3.6% and 0.4% and 1%, respectively. Sensor #4 shows good performance with an RSD of between 1.31% and 5.7%. On the other hand, sensor #2 and sensor #5 show the worst results with an RSD of between 1% and 17% and 0.4% and 60%, respectively. However, statistical significance tests performed using a one-way ANOVA showed no statistically significant differences across the five sensor typologies.

The complete list of the GFs calculated for each sensor at different values of elongation is reported in Appendix A—Table A2. Briefly, sensor #1 and sensor #3 have good GF values, achieving 2.86 and 2.16 at 25% of strain, respectively. At higher values of strain, the GFs decrease, reaching 0.17 and 0.34 at 100% of strain, respectively. Sensor #2 has a decreasing resistance value for strain values of 25% and 50% and an increasing resistance value for strains above 50%. Sensor #4 has negative GF values and thus shows a decrease in the resistance values with increasing strain. Sensor #5 shows good GF values in the range between 0% and 25%. In the literature, good values of GFs for metallic foils are around 2, while for silicone-elastomer structures, the measured GF reduces to 0.23 [41]. Considering this, the results obtained for sensor #1 and sensor #3 are in line with the literature data, and these sensors show good results for low strain. To evaluate the statistically significant differences between gauge factor values at different levels of strain, a one-way ANOVA was performed, followed by post hoc Tukey tests. At 25% and 50% strain, *p*-values of 0.0008 and 0.0019, respectively, were obtained, indicating statistically significant differences. Post hoc Tukey testing revealed significant differences between

- Sensor#1 and sensors #2 and #4;
- Sensor #2 and sensor #3;
- Sensor #3 and sensor #4.

At 75% strain, a *p*-value of 0.0019 was obtained, confirming a statistically significant difference across sensors. Post hoc Tukey testing showed significant differences between

- Sensor #1 and sensor #4;
- Sensor #3 and sensors #4 and #5.

At 100% strain, a *p*-value of 0.00005 was achieved, again indicating statistically significant differences across sensors. Post hoc Tukey testing identified significant differences between

- Sensor #1 and sensors #3 and #4;
- Sensor #2 and sensors #4 and #5;
- Sensor #3 and sensors #4 and #5.

### 3.2. Durability Test

**Figure 6:**
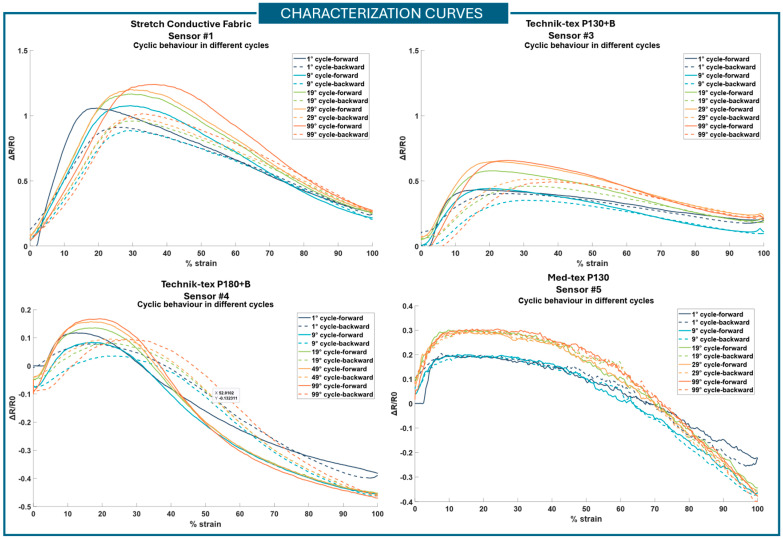
Graphics of the characterization curves for sensors #1 (Stretch Conductive Fabric), #3 (Technik-tex P130+B), #4 (Technik-tex P180+B), and #5 (Med-tex P130). The continuous line is for the upward cycle; the dashed line is for the downward cycle.

The characterization curves of the sensors were obtained from the hundred cycles of the monoaxial tensile test. For the different sensors, the Δ*R*/*R*0 over the 0–100% strain range was plotted for different cycles (1°, 9°, 19°, 29°, 99°). The characterization curves of sensors #1, #3, #4, and #5 are reported in Figure 6. Sensor #2 was removed from the analysis due to a highly non-repetitive behavior, which led to a high drift (0.105 ± 0.027). However, for the completeness of the analysis, the time-course of Δ*R*/*R*0 is reported in Appendix A—Figure A2. It is worth mentioning that, from this plot, the position change from upward to downward cycles cannot be uniquely identified due to its unstable behavior. Considering its behavior, sensor #2 was excluded from the following analysis.

In Table 2, the drift values, max hysteresis error, and operating range for the different sensors are reported. Sensors #1, #3, and #5 show the lowest values of drift. To compare the drift values across sensors, a one-way ANOVA test was performed, yielding a *p*-value of 0.0067. Furthermore, post hoc Tukey testing revealed statistically significant differences between sensor #3 and sensor #4 and between sensor #5 and sensor #4. All the sensors show low hysteresis error. Sensor #3 is the one with the lowest hysteresis error, while sensor #1 is the one with the highest hysteresis error. However, for sensors #3, #4, and #5, the maximum hysteresis error increases with the increasing number of cycles. A one-way ANOVA was performed by comparing the maximum hysteresis errors calculated for the 1st cycle and the 99th cycle. A *p*-value of 0.0048 was achieved when considering the errors calculated for the first cycle. From the post hoc Turkey test, the mean of sensor #1 was significantly different from that of all the other sensors. For the 99th cycle, no statistically significance difference was found across sensors. Regarding the operating range, sensor #1 is the one presenting the widest operating range, between 0 and 30% of strain. The other sensors show a narrower operating range. In this analysis, the first monotonic part of the characterization curves was considered to define the operating range. However, it should be possible to also consider the second monotonic part of the curves by pre-stretching the sensors. For the operating range, a one-way ANOVA statistical test was performed for both the 1st and 99th cycles. A statistically significance difference was identified, with a *p*-value of 0.005 and 0.0002, respectively. In the post hoc Turkey test, for the first cycle, a statistically significance difference was found between

- Sensor #1 and sensors #4 and #5;
- Sensor #3 and sensor #4.

**Figure A2:**
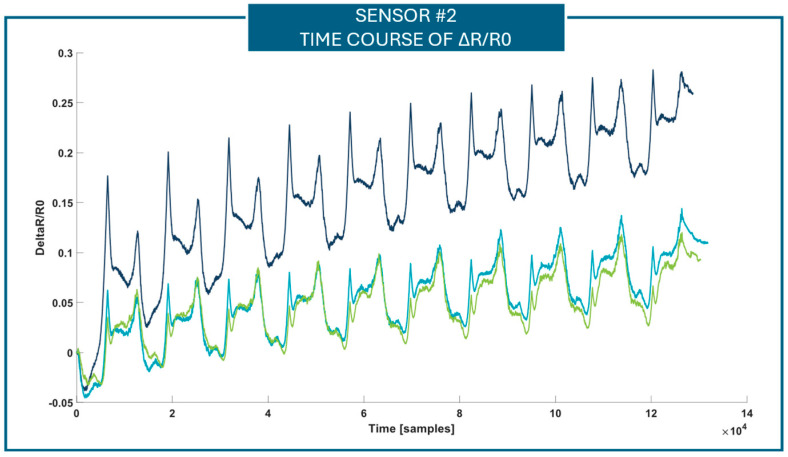
Normalized resistance value vs. time. Blue line: first repetition of 10 cycles. Light blue line: second repetition of 10 cycles. Green line: third repetition of 10 cycles.

For the 99th cycle, a statistically significance difference was found between sensor #1 and sensors #3, #4, and #5. Images before and after the durability test were compared, but no macroscopic differences were found. Only sensor #2 showed macroscopic differences on the surface when stretched over multiple cycles. Rayon fibers have a tendency to change their configuration over time, both breaking and disassembling. The sensor output behavior shown in Figure A2 (in Appendix A) is probably also due to this morphological modification.

## 4. Discussion

All the sensors’ characterization curves (Figure 6) show a non-monotonic behavior; thus, the use of the sensors should be limited to the first monotonic portion of the curve (before the slope change) or to the second one, if a pre-stretch is applied. To understand the phenomena underlying this behavior, further analyses were carried out. Sensor #1 showed good stability and durability properties, and it is the one showing a wider operating range (Table 2); it is the only one to achieve a range of 0–30% of strain. Based on our specifications and the sensors’ behavior, sensor #1 is the most appropriate for integration into our sensorized high-fidelity physical simulator. Thus, it was selected to carry out a complete analysis to investigate the underlying non-monotonic phenomenon.

**Figure 7:**
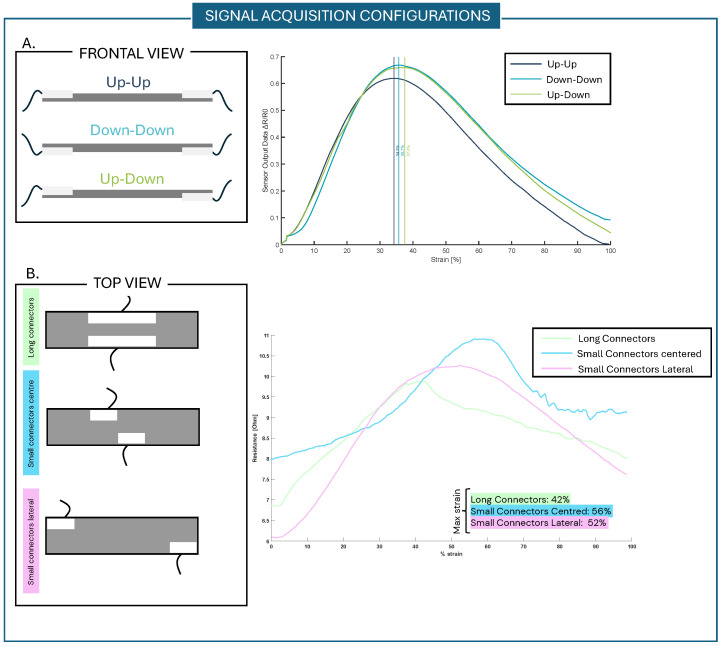
Different signal acquisition configurations. (**A**) The connector configurations were used to understand the behavior and conduction paths between interlayer structures of the sensors. In the three configurations, the sensors respond with similar output behavior. (**B**) The connector configurations were used to understand the behavior and conduction path within surface braid-like structures. By placing the connectors in these positions, it is possible to shift the curve peak and thus increase the operating range of the sensor. A larger shift happens with the small connectors’ lateral configuration.

First of all, to evaluate the silicone substrate’s influence on sensor behavior, tests on the fabric only were conducted. As shown in Figure 7A—up–up configuration—the sensor behaves the same even without the silicone substrate. This non-monotonic behavior could depend on the material used to realize the fabric or on the fabric’s structure, i.e., interaction between the different braid-like structures.

**Figure A3:**
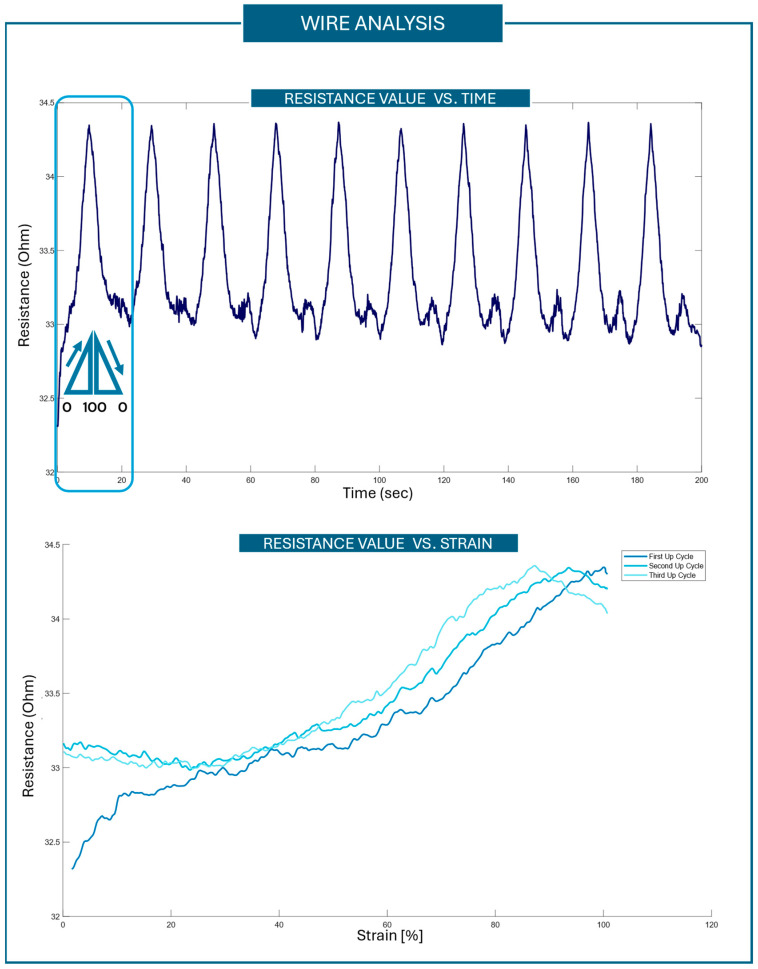
Analysis of conductive wires: TOP: Resistance value vs. time during the stretching cycles is reported. The wire has a monotonic behavior in the considered strain range (0–100%). BOTTOM: the resistance values over strain of the first three up cycles are depicted.

To evaluate the effect of the basic components of the conductive fabrics, tests on conductive wires were performed. Monoaxial tensile tests between 0% and 100% of strain were carried out. The conductive wires show a monotonic behavior across the whole range of strain (Figure A3—in Appendix A). Considering this, the material itself is not the cause of the sensor’s non-monotonicity. Instead, the interaction between the different fibers appears to be responsible for the non-monotonic behavior observed when stretching the conductive fabrics.

**Figure 8:**
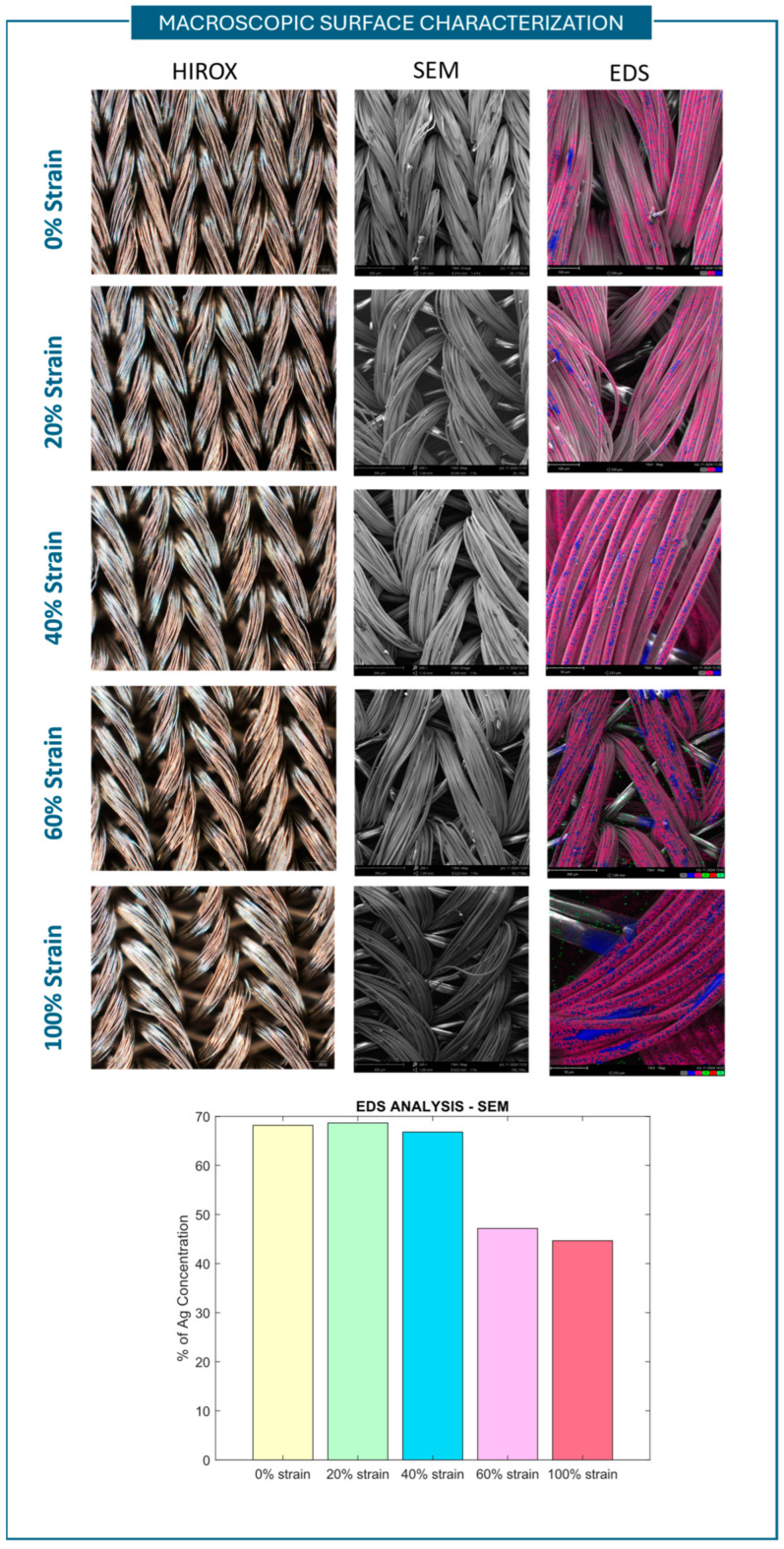
Macroscopic surface characterization conducted for sensor #1. For 0%, 20%, 40%, 60%, and 100% of strain, Hirox images (first column), SEM images (second column), and EDS (Energy-Dispersive X-ray Spectroscopy) analysis (third column) are reported. Hirox images were taken at 140×, SEM images were taken at 190× and 245×, and EDS analysis was carried out with a 15 kV source. In the EDS analysis images, the pink surfaces represent the silver element (Ag). The histogram shows the percentage of Ag in the samples stretched at different values of strain.

To analyze the fibers’ configuration and macroscopic behavior at varying values of strain, i.e., fiber arrangements, Hirox microscope images were taken while stretching the sensor using a linear stage with constant velocity. In Figure 8—first column—the images taken at 0%, 20%, 40%, 60%, and 100% of strain are shown. When the strain exceeds an elongation of 20%, the fiber braids begin to separate, creating more space between them. This could alter the current flow between the braids, prompting further analysis.

To evaluate the effect of the interlayer current flow through the sensor, different signal acquisition configurations were tested. The samples were tested through a monoaxial tensile test (0–100% of strain) using the Instron machine. Firstly, the standard configuration with both the connectors on the top sensor surface was considered (up–up configuration); secondly, the connectors were positioned on the bottom surface of the sensor (down–down configuration). Lastly, one was placed on the top surface of the sensor and the other on the bottom one (up–down configuration), as in Figure 7A. In these three configurations, the sensor mechanical behaviors show characteristic curves where the slope change peaks appear at 34.2%, 35.7%, and 37.5% of strain, respectively. This means that, considering the up–down configuration results, it is possible to state that the current flow passes through the different sensor braids and across the interlayers of the sensor fabric.

Moreover, the obtained results have demonstrated that connectors can be placed both on the top and bottom sensor surface without changing the sensor behavior or characteristic curve. Based on the application needs, it should be possible to choose the best signal acquisition configuration. To understand what happens regarding the current flow onto the sensor surface when the sensor is stretched and the fiber braids separate, other connector configurations were taken into account, as shown in Figure 7B. In particular, to understand whether the characteristic curve is influenced by differences in the current flow onto the sensor surface, three signal acquisition configurations were tested. Sensors with a long-connector configuration; a middle, centered small-connector configuration; and a lateral small-connector configuration were realized and tested. The results showed that the current still flows between the braid-like structures when they separate; in fact, considering the results of the centered small-connector and lateral small-connector configurations, the signal can still be acquired. In addition, by placing the connectors in different configurations, it is possible to shift the change in the slope peak, moving it to the right. The long-connector configuration characterization curve shows a peak at around 42% of strain; the middle, centered small-connector configuration presents a sensor curve inversion point at around 56% of strain; and the lateral small-connector configuration shows a change in the slope at 52% of strain. Considering these results, by placing the connectors in different positions, the conductive paths between fibers can still be captured, and this effect can be used to increase the operating range of the sensor.

Furthermore, an SEM was used to acquire sensor surface images at different values of elongation and also to conduct EDS (Energy-Dispersive X-ray Spectroscopy) analysis. EDS is a non-destructive analytical technique in which the sample is irradiated with electrons, resulting in the emission of X-rays specific to the elements present on the surface. The energy emissions are translated into spectral peaks of variable intensity, resulting in a spectrum profile which identifies the different elements available on the sensor sample surface [42]. The images obtained and the atomical elements recorded are shown in Figure 8—second and third columns. In all sensor # 1 samples, silver was found. The weighted concentration of Ag for each sample was then recorded, and the different samples’ concentration is shown in the histogram at the bottom of Figure 8. The Ag presence on the sample surfaces decreases with increasing strain after 40%. By considering these results, we can state that the sensor is anisotropic in terms of conductive elements on its surface, and thus, the resistivity of the material changes during the stretching tests.

**Figure 9:**
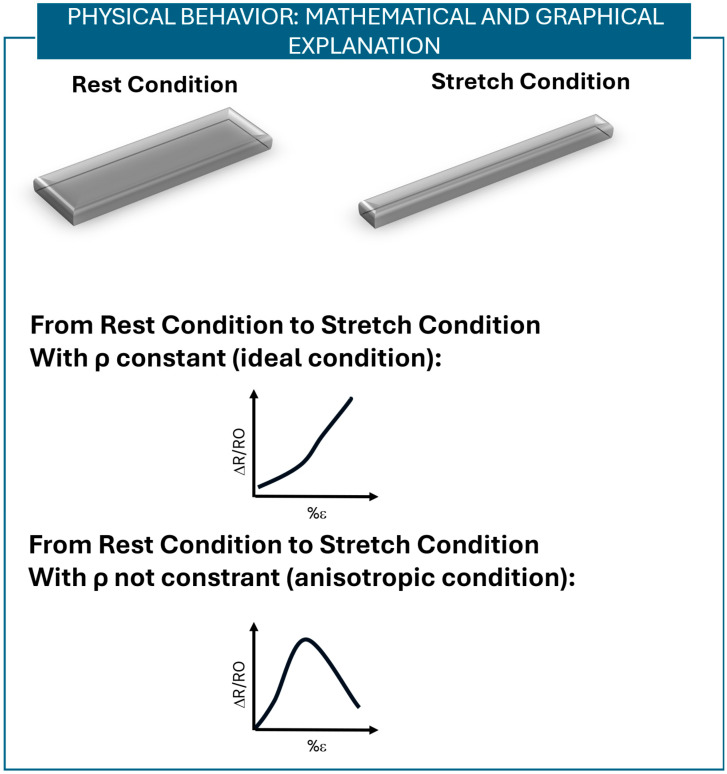
Mathematical and graphical explanation of the sensor’s non-monotonic behavior. In our specific case, due to the sensor’s configuration and construction, *ρ* should not be considered as a constant. In particular, at increasing strain values, the number of conductive paths on the sensor increases; the conductivity (*σ*) thus increases, reducing the resistivity (*ρ*) of the sensor and thus the resistance value (*R*).

By considering all the results obtained, the behavior of the sensor can be shown in mathematical and graphical terms, as reported in Figure 9. Resistance (*R*) is given by

(4) $$R=\rho \;\frac{l}{A}$$

When subjected to strain, the length (*l*) of the sensor increases and the area (*A*) decreases; thus, by considering a constant ρ (intrinsic to the material properties) related by

(5) $$\rho =\frac{1}{\sigma }$$

to the conductivity *σ*, *R* should increase. However, in our specific case, *ρ* should not be considered as constant. The material properties change due to the high anisotropic distribution of the braid-like structures and sensor fibers. When the sensor is stretched, especially after a certain percentage of length, the conductive paths between fibers increase, and the interlayer paths start playing a role in the conduction behavior of the sensor (Figure 8 and Figure 9). The *R* starts decreasing when a certain value of strain is reached due to a decrease in ρ. The decrease in ρ is caused by an increase in the conductivity σ of the sensor due to possible conductive paths and interlayer conduction. In more detail, according to the percolative model with a tunneling effect [43], the anisotropic geometry of the sensor explains its non-monotonic behavior. In the initial portion of the characterization curve (before the slope change) (Δ*R*/*R*_0_ vs. % ε), the number of conductive paths decreases as the fibers separate along the direction of applied strain. However, due to the sensor’s anisotropy, fibers in the transverse direction move closer, promoting the formation of additional conductive paths through the tunneling effect. This results in increased overall conductivity and, consequently, a decrease in resistance.

## 5. Conclusions

In this work, we proposed a comprehensive methodology to characterize fabric-based resistive stretching sensors, from both static and dynamic perspectives, for future integration into high-fidelity physical simulators of vessels for surgical training.

Five sensor types made from five commercially available conductive fabrics were characterized. Stability tests were conducted to evaluate sensor behavior when kept under constant strain and the gauge factors. Durability tests were carried out to study sensor behavior when subjected to fatigue. Furthermore, an analysis of the characteristics of the involved sensor fibers was carried out to strengthen the characterization. Due to the expansion of flexible sensors, the fabrics were designed to be integrated into soft silicone structures to realize flexible sensors.

Considering the results of the stability test, sensor #1 and sensor #3 showed low relative standard deviations across different strain levels. The gauge factors of these sensors were also in line with the literature values, showing good sensitivity at lower strain values. Sensor #2 and sensor #5, instead, showed higher values of RSD and a less stable behavior. The results of the durability test showed that sensors #1, #3, and #5 had the lowest drift values. All sensors had low hysteresis errors, with sensor #3 having the lowest. Regarding the operating range, the sensor with the widest operating range is sensor #1, with an operating range of up to 30% of strain. Digital microscope images were used to analyze macroscopic changes on the surfaces of the sensors. According to these images, only sensor #2 showed morphological changes that contribute to its overall instability.

All the sensors show a non-monotonic behavior when stretched over a 0–100% strain range. Based on our application specifications, the only sensor showing high stability, good sensitivity at low strain values, and an operating range of between 0% and 30% of strain is sensor #1. Thus, it was selected for a deeper analysis to investigate the underlying behavior. The non-monotonic behavior could depend on the fabric’s material or on the fabric’s structure. To investigate the influence of the material, elastic conductive wires were tested, and we found that they show a monotonic sensor output behavior in the 0–100% strain range. To evaluate the effect of the braid structure of the sensor, surface images were taken using a Hirox microscope to understand how the fibers spread when subjected to strain. At increasing values of strain, the braids tend to separate; thus, different signal acquisition configurations were tested, and we showed that different conductive paths exist between the sensor braids and the interlayers of the sensor. In addition, these new connector configurations allowed us to achieve higher operating ranges; thus—depending on the application—different signal acquisition configurations can be chosen. SEM images and EDS analysis showed that at increasing values of strain, the percentage of silver decreases. The non-monotonic behavior of the sensor can be explained through the anisotropy of the sensor structure. This leads to an increase in the conductive paths when the sensor is stretched and thus a decrease in the sensor’s resistivity and resistance when subjected to strain, as reported in Figure 9.

In the context of surgical training and simulation, both the working and experimental conditions are highly controlled, as the training sessions are conducted within an operating room equipped with the da Vinci Surgical System. This environment maintains stable temperature and humidity levels. Therefore, in our specific application, the sensor is not subjected to significant variations in environmental conditions, and dedicated temperature and humidity stability tests were excluded from the characterization process. Nonetheless, should the sensor be considered for other applications involving variable environmental conditions, targeted characterization tests will be designed and conducted accordingly.

To conclude, the study provides a comprehensive methodology for characterizing fabric-based resistive stretching sensors to identify the best sensor among the ones tested for developing sensorized high-fidelity physical simulators of vascular structures. Although sensor integration with soft silicone structures is not straightforward, conductive fabrics represent a smart and low-cost solution for sensorizing soft structures without altering their original mechanical properties and shape.

## Figures and Tables

**Table 2:** Drift, max hysteresis error, and operating range. The sensors (#1, #3, #5) showing the lowest drift are highlighted in light yellow. The sensor (#1) showing the highest operating range is highlighted in light green.

Drift, Max Hysteresis Error, and Operating Range					
		Sensor #1	Sensor #3	Sensor #4	Sensor #5
Drift	(mean ± std)	(0.050 ± 0.010)	(0.027 ± 0.024)	(0.090 ± 0.008)	(0.033 ± 0.020)
Max hysteresis error 1st cycle	Sample 1	0.35	0.13	0.05	0.12
	Sample 2	0.30	0.03	0.09	0.05
	Sample 3	0.19	0.06	0.10	0.13
Max hysteresis error 99th cycle	Sample 1	0.25	0.35	0.16	0.20
	Sample 2	0.33	0.19	0.15	0.06
	Sample 3	0.30	0.25	0.15	0.26
Operating range 1st cycle	Sample 1	0–19.5	0–17.0	0–12.5	0–17.0
	Sample 2	0–20.8	0–20.5	0–8.6	0–9.3
	Sample 3	0–22.4	0–18.6	0–12.1	0–14.7
Operating range 99th cycle	Sample 1	0–35.8	0–25.0	0–19.2	0–22.4
	Sample 2	0–34.6	0–25.0	0–17.0	0–24.0
	Sample 3	0–31.7	0–27.8	0–17.6	0–19.8

## Data Availability

The raw data supporting the conclusions of this article will be made available by the authors on request.

## Appendix A

**Table A1:** Means and standard deviations of the normalized resistance values at each value of elongation.

Means and Standard Deviations in the Stability Test					
25% of strain					
	Sensor #1	Sensor #2	Sensor #3	Sensor #4	Sensor #5
Sample 1	0.68 ± 0.01	−0.074 ± 0.002	0.540 ± 0.002	−0.106 ± 0.003	0.30 ± 0.01
Sample 2	0.716 ± 0.003	−0.082 ± 0.002	0.434 ± 0.002	−0.114 ± 0.002	0.003 ± 0.013
Sample 3	0.186 ± 0.002	0.036 ± 0.002	0.491 ± 0.002	−0.128 ± 0.005	0.174 ± 0.009
50% of strain					
	Sensor #1	Sensor #2	Sensor #3	Sensor #4	Sensor #5
Sample 1	0.55 ± 0.02	−0.054 ± 0.002	0.493 ± 0.003	−0.171 ± 0.004	0.29 ± 0.01
Sample 2	0.575 ± 0.003	−0.022 ± 0.003	0.438 ± 0.002	−0.149 ± 0.003	−0.10 ± 0.06
Sample 3	0.075 ± 0.001	0.027 ± 0.005	0.506 ± 0.002	−0.175 ± 0.01	0.05 ± 0.01
75% of strain					
	Sensor #1	Sensor #2	Sensor #3	Sensor #4	Sensor #5
Sample 1	0.342 ± 0.008	0.054 ± 0.003	0.389 ± 0.003	−0.250 ± 0.004	0.15 ± 0.01
Sample 2	0.34 ± 0.01	0.086 ± 0.015	0.402 ± 0.003	−0.202 ± 0.004	−0.24 ± 0.04
Sample 3	−0.058 ± 0.002	0.17 ± 0.02	0.503 ± 0.005	−0.242 ± 0.004	−0.161 ± 0.007
100% of strain					
	Sensor #1	Sensor #2	Sensor #3	Sensor #4	Sensor #5
Sample 1	0.172 ± 0.003	0.241 ± 0.003	0.340 ± 0.002	−0.360 ± 0.004	−0.086 ± 0.007
Sample 2	0.169 ± 0.002	0.290 ± 0.003	0.348 ± 0.002	−0.305 ± 0.004	−0.274 ± 0.001
Sample 3	−0.1609 ± 0.0008	0.397 ± 0.004	0.463 ± 0.003	−0.322 ± 0.009	−0.361 ± 0.004

**Table A2:** Gauge factors calculated for each sensor sample at the different values of strain.

Gauge Factor					
25% of strain					
	Sensor #1	Sensor #2	Sensor #3	Sensor #4	Sensor #5
Sample 1	2.74	−0.30	2.16	−0.42	1.19
Sample 2	2.86	−0.33	1.73	−0.46	0.01
Sample 3	0.74	−0.14	1.96	−0.51	0.70
50% of strain					
	Sensor #1	Sensor #2	Sensor #3	Sensor #4	Sensor #5
Sample 1	1.09	−0.11	0.99	−0.34	0.57
Sample 2	1.14	−0.04	0.89	−0.30	−0.20
Sample 3	0.15	0.05	1.01	−0.35	0.10
75% of strain					
	Sensor #1	Sensor #2	Sensor #3	Sensor #4	Sensor #5
Sample 1	0.46	0.07	0.52	−0.33	0.20
Sample 2	0.45	0.12	0.54	−0.27	−0.32
Sample 3	−0.08	0.23	0.67	−0.32	−0.21
100% of strain					
	Sensor #1	Sensor #2	Sensor #3	Sensor #4	Sensor #5
Sample 1	0.17	0.24	0.34	−0.36	−0.09
Sample 2	0.17	0.29	0.35	−0.30	−0.27
Sample 3	−0.16	0.40	0.46	−0.32	−0.36
